# Legislation Concerning Tuberculosis

**Published:** 1899-04

**Authors:** 


### Legislation Concerning Tuberculosis.

A natural sequence of the general acceptance of the belief in the infectious character of tuberculosis, is a public demand for State intervention to prevent its dissemination. It is also to be expected that many measures of doubtful efficacy will be proposed, along with those of unchallenged utility.

This statement applies to the numerous measures relating to tuberculosis, which have been already introduced into the legislature at Albany, to some of which we wish to call the attention of our readers. The most important of these, perhaps, is Senate bill No. 12 (Mr. Davis), providing for the establishment of a State hospital in the Adirondack region for the treatment of incipient tuberculosis. There are many reasons why State aid should be furnished for those unfortunates who are not otherwise provided for, but this proposed law provides for pay cases as well as the poor, and we believe the time has not yet arrived for undertaking an enterprise which will certainly involve a larger expense than that required to maintain the pauper lunatics of the State. There are over 20,000 lunatics in State hospitals. The number of consumptives is very much larger than that, and the legislature must ultimately provide for all who may apply.

As a matter of urgent sanitation, the isolation of *advanced cases* is of the greatest possible importance as a prophylactic measure. Existing general hospitals will not receive them (and it is proper that they refuse them), and yet through their presence in their homes they are a menace to myriads of people whose services are of great value to the State. Incipient cases may be safely treated in existing institutions and even in their own homes.

At the risk of being charged with a personal motive we submit the general proposition, that the State Board of Health, which is required by law to limit the spread of tuberculosis in every possible way, should be entitled to at least as much of a voice in any enterprise of the character proposed as the State Forest Commission, which the bill does not overlook. Is it not a little out of the natural order that a great State hospital should be located, erected, equipped and managed by a commission containing no representative of the medical profession? We have no doubt Governor Roosevelt would supply the omission in the bill, if enacted, by appointing a medical member, but it should be a statutory provision. We would prefer to have the act amended so as to provide a place for the infectious (advanced) cases, if such an institution is to become a part of the "State care" system.

Another bill (Assembly bill No. 351), introduced by Mr. Henry, provides that cities of the first class (New York and Buffalo) may establish and maintain hospitals for the treatment of pulmonary tuberculosis outside of their corporate limits.

The leading idea of this proposed enactment is to remove infectious cases to a more salubrious as well as a safer location than can be found within the confines of these great cities. The enactment of its provisions into law would be a decided advance in sanitation, and also decidedly humanitarian in its operation.

The health authorities of these municipalities would certainly be relieved of a perplexing problem, and the large communities interested protected as well. The legislature should pass the bill without delay.—*Medical Review of Reviews.*

